# A Common Symptom Complex Explained

**Published:** 1920-12-11

**Authors:** 


### INFANTILE ACIDOSIS.

### A Common Symptom Complex Explained.

It is a well-recognised fact among those whose duties bring them into contact with children's ailments that in the course of a gastro-intestinal disturbance of infancy, so called "toxic" symptoms may develop, and on occasions appear to be even a direct cause of death. But the true mechanism of this state is not well understood. The profession is indebted to Dr. R. H. Dennett, of the New York Post-Graduate School, for a particularly clear explanation of acidosis, in the revised edition of his book* on *Simplified Infant Feeding.*

As he explains at the outset, it is a common misconception to consider acidosis a cause, or as of itself a disease entity, when it is in reality nothing more than a symptom complex. Essentially the state is defined as a reduction of the sodium bicarbonate of the blood below the normal level. This generally occurs as a super-imposed condition, which may in itself threaten life, but in no way serves as a real explanation of the disease. And acidosis must be clearly distinguished from toxæmias of gastro-intestinal origin occurring in infants, the true etiological factors being the toxic agents of bacterial origin.

THE PATHOLOGY.

But we shall return to this later. In acidosis the reaction (alkalinity) of the blood is altered, whereas normally there is nothing in the animal economy which remains more constant than this reaction. The exact physiological process need not be detailed, but, if the acid-alkali balance is permanently disturbed, a definite train of symptoms will become at once apparent.

Here we need mention only those most obvious at the bedside. There is rapid and markedly deeper breathing. The child shows a remarkable tolerance for sodium bicarbonate given by the mouth; the usual dose of forty-five grains is sufficient to render the urine of normal infants alkaline, whereas in acidosis five times the amount may be required. Typically acetone and diacetic acid may be excreted, and in severe cases stupor or coma may develop. Also the excretion of urine becomes diminished, and often at this stage contains albumin and renal casts. The most remarkable and certain sign, however, is the rapid improvement, especially reduction of the hyperpnœa, after correction of the acidosis by administration of alkali.

Better to appreciate how the above changes develop, it is essential to realise how acids are normally eliminated from the body, and hence the correct alkalinity of the blood preserved. There are three ways: (1) Gaseous carbonic acid is eliminated through the lungs. (2) The fixed acids are excreted through the kidneys. (3) Certain acids, such as phosphoric, are got rid of *via* the intestines. If normal or abnormal influences add acids to the circulation they are at once neutralised by the so-called "buffer" substances in the blood plasma (sod. bicarbonate and certain proteins), also by similar substances in the blood cells and tissues of the body and by increased activity of the excreting organs. When, however, other acid substances have entered the blood-stream, and united with the available alkaline protectors, there develops a decreased carbonic acid combining power in the plasma and a corresponding attempt at increased pulmonary ventilation—*i.e.*, hyperpnœa.

CAUSAL CONDITIONS.

Therefore it is obvious that there will probably be more than one infantile disease likely to disturb the alkalinity of the blood, and so produce acidosis. Dr. Dennett considers that in practice the most important are, in order of frequency: (1) Cyclic vomiting; (2) starvation; (3) post-anæsthesia; (4) diabetes; (5) uræmia; (6) intestinal toxæmia. Obviously, this is not intended to be a complete list, but the sequence of events in each is instructive. The mechanism in an acidosis is practically always the same. In diabetes the root cause is a direct perversion of metabolic processes with overproduction of acid substances; in uræmia there is inability on the part of the kidney to get rid of the acid products of a normal metabolism. In toxæmia of alimentary or other origin the etiological causes are toxic agents acting on the metabolic or excretory functions or both. In starvation, which is practically what a number of infantile vomiting disorders indirectly amount to, the acidosis often noted may be due particularly to a relative absence of carbohydrates from the diet, in the same way that the condition is produced in acute infections where there is difficulty in giving sufficient food of any kind.

MEANS OF PREVENTION.

As acidosis is not a disease in itself, it is obvious that means of prevention are of first importance. Osler has summarised the essential measures as follows: (1) The giving of large amounts of water. (2) The administration of carbohydrate. This may be by mouth or by bowel, as by the use of a 2 to 5 per cent. solution of glucose. (3) The giving of soda-bicarbonate by mouth, bowel, or even intravenously until the urine is alkaline. The last is particularly important in dealing with the earliest stages in children—and it is also found that administration of calcium, potassium and magnesia, as well as sodium salts, may be markedly advantageous. When the condition is once established, apart from removal of the underlying cause, the above methods still hold good. But, as already explained, with children the secret of success lies in anticipating rather than treating a severe acidosis.

So much for acidosis in general and the commoner of its causes; but Dr. Dennett puts forward an interesting explanation of an acute state, simulating acidosis, and caused by bacterial alimentary toxæmia, and it is at least based upon more accurate

* London: J. B. Lippincott. Price 21s. net.

**Infantile Acidosis**—(*continued*).

bacteriological observations than are common in the majority of text-books. A considerable group of anærobes commonly found in the intestine are, in certain circumstances, capable of producing not only powerful hæmolytic toxins, but also a widespread cloudy or fatty degeneration of parenchymatous cells. Indirectly this may result in true acidosis, but if these toxins are absorbed into the general circulation in sufficient quantities they may of themselves cause death within relatively few hours.

"It is to be emphasised that this group of anærobes are not putrefactive in their main action, though it has to be confessed that they best form toxins in the presence of an alkaline reaction and in a medium that is red in decomposing proteins. Typical members of this group of organisms are certain strains of *B. Welchii, the Vibrion septique*, and *B. œdema tiens*. These organisms do not cause putrefaction. They break down sugars rapidly with the formation of butyric acid."

We know that a number of true putrefactive organisms, of which *B. spirogenes* (Metchnikoff) is the type, can produce an extremely acute intestinal infection, and, with the above, are all common in the upper layers of the garden soil, in dust, and putrescent material. Also they will all grow well in milk, and some are found in small numbers pre-existing in the intestinal tract. It is this particular type of infection, according to the author, that lies at the root of the majority of infantile toxæmias of alimentary origin which result in symptoms of acidosis.

This is an interesting suggestion when viewed in connection with the blame which is customarily laid upon the coliform group of organisms for so many alimentary disturbances in the child, and although the above possibilities are fully recognised in pathological practice, it is a point worth bearing in mind in connection with the practical question of "clean milk" production. The anærobic bacteria are, it is true, not all spore-bearing organisms, but some of the above most certainly are, and their destruction by "sterilisation" processes involves corresponding provision.

Such details form, however, something of a digression from our consideration of infantile acidosis as such, and, to return, one or two further points in its actual treatment may be considered. In the majority of instances it is advisable to clean out the whole of the alimentary tract in the shortest possible length of time. Dr. Dennett advises a thorough and painstaking washing-out of the stomach and large intestines. A very little castor oil, a quarter to half an ounce, may be left in the stomach, and the large intestine may be left full of 5 per cent. sodium bicarbonate. Calomel is too slow in its action to be an ideal cathartic in these cases, and magnesium sulphate tends further to deplete a body already badly in need of fluids. Apart from general measures to promote excretion, normal saline may be injected intravenously or even intraperitoneally in urgent cases. Other methods of reducing alkalinity have already been described, but it is a point to remember in performing subcutaneous or intraperitoneal operations that it is essential to sterilise the sod. bicarbonate *dry*, to prevent, in both cases, the formation of the carbonate which is often intensely irritating and certain to cause sloughing or necroses. Intravenously, which is the best method, this risk is not important, for not only is incorporation of the alkaline into the body fluids effected at once, but neutralisation of the acid products is far more rapid. Unfortunately, it is not, however, always easily carried out in the instance of very small children.

The rationale of the alkaline treatment having been dealt with, there remains only the question of diet. Starvation for the first twelve to twenty-four hours is probably advisable. Afterwards the first foods allowed should be carbohydrates—*i.e.*, glucose and starches. Well-cooked cereal gruels are excellent, and, given at first in small quantities, the amount is rapidly increased. It is a common mistake to return to a milk diet far too early, although in a successful case this is obviously the ultimate objective; but in no case should it be persisted in until the child's metabolism is ready to deal with the more complicated food substances thereby involved.

